# A Case Report of Compassion Focused Therapy (CFT) for a Japanese Patient with Recurrent Depressive Disorder: The Importance of Layered Processes in CFT

**DOI:** 10.1155/2018/4165434

**Published:** 2018-10-17

**Authors:** Kenichi Asano, Eiji Shimizu

**Affiliations:** ^1^Department of Psychological Counseling, Faculty of Human Sciences, Mejiro University, Japan; ^2^Research Center for Child Mental Development, Chiba University, Japan

## Abstract

Major depressive disorder is a common mental health problem around the world. To treat depression, cognitive behavioural therapy is highly recommended by some guidelines. However, there are reports pointing out the existence of patients who do not respond to cognitive behavioural therapy because of dissociation between thoughts and experiences. To treat such patients, compassion focused therapy was developed, but there are no reports of compassion focused therapy for Japanese patients. This report presents a case of compassion focused therapy for a Japanese female with major depressive disorder and suicidal feelings. After receiving compassion focused therapy, the patient recovered and began to have social interaction with others again. This case suggested the importance of psychoeducation, exercises involving compassionate images and breathing, and a compassionate relationship in conducting compassion focused therapy.

## 1. Introduction

Major depressive disorder (MDD) is a common mental health problem all over the world [1]. More than 40% of mental illnesses are classified as MDD and this rate is twice that of anxiety disorders [2]. So, it is natural that MDD is beginning to be regarded as a social issue [3].

To treat MDD, cognitive behavioural therapy (CBT) is recommended by major treatment guidelines [4, 5]. The effectiveness of CBT is meaningful, and significant in treating depression [6]. However, some patients tend to show difficulties in cognitive restructuring, which is the main strategy of CBT [7]. Gilbert pointed out that some patients cannot experience safety or comfortable emotions even though they could generate adaptive thoughts, and argued that this is related to the tone of voice [8]. Such tendencies are seen in patients with high self-criticism and shame, because they either cannot or find it extremely difficult to be compassionate toward the self. This hypothesis is supported by epidemiological reports [9, 10], and therefore compassion is a key factor in treating such patients.

To treat such patients Gilbert developed compassion focused therapy (CFT) [8]. CFT is considered a new psychotherapy with potential for treating patients with high self-criticism and shame. An early systematic review and early meta-analysis investigated CFT and found that it can be a useful treatment for chronic psychological problems [11, 12]. However, there are no reports of CFT for Japanese individuals even though Japanese culture has been called a “shame culture” by cultural anthropologists [13]. In this report, we report on how CFT was effective for a Japanese MDD patient with high shame and self-criticism. The case report was approved by the ethical committee of the graduate school of medicine, Chiba University. 

## 2. Case Presentation

### 2.1. Case

Lisa (not her real name) was a female aged in her 30s. She was referred from her primary doctor to our CBT Center at Chiba University Hospital.

### 2.2. Major Complaint

Her major complaint was suicidal feeling and depressed mood.

### 2.3. Medical History

Lisa had no prior problems with mental health.

### 2.4. Family History

Lisa's family consisted of her father, mother, grandmother, and younger brother. There were no histories of psychiatric disorders in her family.

### 2.5. Developmental and Social History

Lisa had not had any psychiatric or psychological problems during her childhood, adolescence, or youth. She entered university after graduation from high school. She studied social welfare at university. After graduation from university, she began working in a nursing home as a social worker for 9 years. After that, she began working at a city office as a public assistance consultant for 3 years.

### 2.6. History of Present Illness

Lisa was referred in September 2017 to the CBT Center at Chiba University Hospital. Two and a half years ago, she had consulted a psychiatrist because she overdosed with sleeping pills. She was diagnosed with major depression and received medication for 6 months and took a leave of absence. During that period, she had often gone out to do things that she wanted to do. From September 2015, she started to work part-time at a bakery. Even though she hardly worked, she could not do the job well. During that work, she said to herself, “You must do your best because you decided to work in a bakery” and always felt pressured. She gradually began to make mistakes, and thought to herself, “I am stupid, I can't work well”. After her mother became concerned and asked about her mental state, she had become unable to go to work and quit her job (January, 2016). She began to consult her previous psychiatrist and received medication again. In summer 2016, she had recovered and often went out. However, in September 2016, she began to experience suicidal feelings and considered suicide methods. In the beginning of 2017, she continued to think about dying but in September, her mother recommended that she receive CBT and visited our hospital.

She could help her family with housework and agriculture at her family business and maintained an orderly lifestyle. However, she did not contact her friends after becoming depressed.

### 2.7. Present Illness and Assessment

At the first visit as an intake interview, her depressive symptoms were assessed by a structured interview and self-rating scales, as explained below.

#### 2.7.1. Mini-International Neuropsychiatric Interview (MINI).

The MINI is a useful instrument to check for psychiatric problems [14]. The result suggested the possibility of MDD and Social Anxiety Disorder.

#### 2.7.2. Beck Depression Inventory-II (BDI-II)

The BDI-II is the most popular self-rating scale for depression [15]. The BDI-II consists of 21 items rated on a 4-point Likert scale. Patients select the most adequate response for their mental states, ranging from 0 to 3. The Japanese version of the BDI-II has been developed and its scoring is classified from 0 to 13 as minimal, from 14 to 19 as mild, from 20 to 28 as moderate, and from 29 to 63 as severe depressive symptoms. It has been also standardised and shown sufficient reliability and validity [16]. The patient scored 32 points on the BDI-II.

#### 2.7.3. Patient Health Questionnaire-9 (PHQ-9)

The PHQ-9 is a useful self-rating instrument for primary care evaluation of mental problems, and has been standardised by using a huge sample of patient data [17, 18]. The Japanese version of the PHQ-9 was standardised and showed sufficient validity and reliability [19]. Scores of more than 10 points should be considered as clinical depression and referred to professional services. About severity, from 0 to 4 represents absence of depression, from 5 to 9 is minimal, from 10 to 14 is moderate, from 15 to 19 is moderate to severe, and more than 20 is severe. The patient scored 17 points on the PHQ-9 and was evaluated as moderate to severe.

#### 2.7.4. Liebowitz Social Anxiety Scale (LSAS)

The LSAS is the most popular self-rating scale for social anxiety [20]. The LSAS consists of 24 items rated by a 4-point Likert scale. Patients select the most adequate response for their social anxiety from 2 aspects (fear and avoidance). The LSAS Japanese version (LSAS-J) was standardised and showed sufficient validity and reliability [21]. In the LSAS-J, 30 points is considered borderline, and scores from 50 to 70 are classified as moderate, 70 to 90 as severe, and more than 90 as highly severe. The patient scored 88 points on the LSAS-J.

#### 2.7.5. Self-Compassion Scale Short Form (SCS-SF)

The SCS-SF is the short version of the self-compassion scale (SCS) by Neff [22]. The SCS is the most frequently used scale to assess the degree of self-compassion and has been translated into many languages. The Japanese version of the SCS-SF has been standardised and has shown good reliability and validity [23]. The mean score of undergraduate students was 17.39 (SD = 4.21) in the self-compassion factor, 18.38 (5.23) in the self-coldness factor, and 35.01 (SD = 7.06) in the overall self-compassion score. Lisa scored 11 in the self-compassion factor, 17 in the self-coldness factor, and 20 in the overall self-compassion score.

### 2.8. Diagnosis

Lisa had been diagnosed with major depressive disorder by a primary psychiatrist, and in our hospital, the psychiatrist also diagnosed her with major depressive disorder based on the above information.

### 2.9. Case Formulation and Treatment Plan 

Based on symptoms, diagnosis, and history of depression, the therapist had planned to treat her with CFT because Lisa regarded herself as deeply shameful and was self-critical, and these had brought about her suicidal feelings. With respect to social aspect, fortunately, she did not have obvious, external severe stressors. With respect to the biological aspect, she had shown symptoms of lack of energy, psychomotor agitation, and diminished ability to think; however, they were not obstacles to receiving psychotherapy.

With respect to the psychological aspect, because her self-criticism and shame seemed persistent and were barriers to developing a compassionate mind, which created fears of compassion [24], the therapist evaluated that it is difficult to use the techniques to develop a compassionate mind in a direct way since the beginning. Therefore, the therapist planned to start psychoeducation to introduce emotion regulation and theories of CFT, to provide the framework for understanding her painful emotions. Such understanding of painful emotions are regarded as one of the layers of CFT, called compassionate understanding, which can build a foundation of compassionate practice and help the patient to regulate threat-based emotions [25]. Furthermore, her lifestyle was adequate, and treating her emotional problems had priority over treating her behavioural problems. However, after learning how to manage emotions, it seemed that the absence of interpersonal activities needed to be treated. Compassionate behaviour, such as assertive communication, was thought to be a useful choice as a specific intervention. The techniques used in this case are from a text of CFT [8]. To enhance her self-esteem and induce autonomy indirectly, the therapist asked her opinion after suggesting plans or ideas in the all sessions.

To ensure and improve the treatment, the therapist underwent a 3-day workshop on CFT and received supervision from the supervisor of The Compassionate Mind Foundation once per month.

### 2.10. In the First Session of CFT

In the first session, the therapist asked about the history of Lisa's life and chief complaint. She talked about her circumstances and about her suicidal feelings. The therapist reflected her painful emotions and provided psychoeducation about suicidal feelings (suicidal feelings are caused by painful emotions, and she can learn how to cope with them) to show understanding, which is an important aspect of CFT. In addition, the therapist and Lisa talked about how to deal with suicidal feelings. She would promise to come to hospital in the next session without hurting herself.

### 2.11. In the Second Session of CFT

In the second session, Lisa was taught about “what is compassion”, and evolutionary perspectives from CFT theories. She responded to this psychoeducation with tears and stated that when her family had said “It is not your fault” to her before, she could not agree. However, she could understand the reason that it was not her fault from a CFT perspective, and how the impact of the vicious circle was produced by the brain's evolution. After psychoeducation, the therapist introduced mindfulness and soothing rhythm breathing (SRB) to handle her emotions. The patient reported reduced anxiety and self-criticism.

### 2.12. In the Third Session of CFT

Lisa had continued the practice SRB. She said that she had begun to stop ruminating negatively and gradually feel relaxed. However, if she had tried to relax, she had tended to think that “relaxing is lazy”, and “I always need to do something” in her mind. Therefore, the therapist introduced the idea of behavioural activation, the three-circle model (which is a theory about motivational and emotional systems used in CFT), and the role of compassion for human to emphasise the importance of relaxing and performing compassionate activities in life. After that, she said that she wanted to activate the soothing system on one hand, and yet on the other hand, she felt anxiety about having a fun or happy time because she had not worked yet. The therapist assessed that she was still in the stage of gradually laying the foundation for compassion, and used a guided image exercise involving compassionate colour as the next step.

### 2.13. In the Fourth Session of CFT

Lisa reported that she was using honorific words in talking with her family. She said that the reason to do so was that she was not the same rank as other family members after she had developed depression. She stated that she was feeling so sorry for her family and must be seen as fine to avoid darkening the mood. The therapist validated her anxiety and avoidance of emotional expression at home. She answered that she was glad that the therapist had noticed. After discussion about this avoidance, she noticed her belief that “if she stopped working hardly, she would be worthless”. She had also said that if she was not a good child she would be worthless. The therapist provided psychoeducation about the qualities of compassion to help her to understand compassion more deeply. The therapist asked her to record her feelings and thoughts in columns as self-monitoring for generating helpful and compassionate thoughts.

### 2.14. In the Fifth Session of CFT

Recording Lisa's feelings and thoughts was painful work for her but she thought that “I need to face myself even though it is useless”. The therapist introduced an imagery exercise of a safe place as a useful method to activate the soothing system. She could feel kindness, warmth, and safety from the exercise. After that, the imagery exercise of a compassionate self was conducted. At first, she could not imagine a kind or strong self well. However, after she imagined a self with wisdom (showing understanding to her), she began to feel a compassionate self with warmth and strength. On the other hand, she also thought that she had lost her ideal self and felt a little bit of anxiety. The therapist validated her anxiety and emphasised the importance of continuing to practice.

### 2.15. In the Sixth Session of CFT

Lisa found a reduction in negative rumination by using a compassionate self-exercise. In the imagery exercise of compassionate others, she imagined an animation character, Luffy. The image of Luffy encouraged her to proceed to the next step without depressive rumination. The image of a compassionate self was kind and warm for her, and the image of compassionate others was strong for her. In this session Lisa practiced imagery exercises (compassionate self and others) again. In addition, the therapist recommended that she use her compassionate self in the column to generate helpful thoughts.

### 2.16. In the Seventh Session of CFT

Lisa reunited with her friend and had a relaxed time. Even though she felt strong anxiety, she could try it by using compassionate thoughts. She said that she had avoided doing so to avoid revealing her mental problem. However, she could have a good time with her friend and advised her friend to validate herself by introducing CFT exercises. In addition, she hoped to start to seek a part-time job again. Because she reported a distressed episode in which she could not tell her mother her opinion, therefore the therapist introduced the idea of assertion and tried role-play.

### 2.17. In the Eighth Session of CFT

Lisa talked about her mother, who is a demanding and aggressive woman. Her mother always advises her to be more assertive, but she had not yet done so. However, she could ask her mother to check her application letter for work and felt she was gradually becoming assertive. She could pass the employment exam and would start work once a week. She could also contact her other friend who was a colleague of a previous bakery by e-mail and could enjoy the interaction. To cope with anxiety related to work, she could imagine a compassionate self who encouraged her to face her difficulties. The therapist assessed that her interpersonal avoidance was reduced by the encouragement of her compassionate self, so she could easily adapt the strategies the therapist taught.

### 2.18. In the Ninth Session of CFT

Lisa experienced some situations in which she could be assertive with her mother. Even though her mother could not respond to her well, she could consider her mother's perspective without negative attributions or aggressive attitudes. She could also start to work at an adult day care centre. She recalled memories of a previous job at a city office. She experienced being ignored by her boss and attacked by her colleague in the previous job. She noticed that this experience had become a block to being either assertive or compassionate with herself. When the therapist asked her “What will you do now if the same thing occurred?”, she answered that she would say “You are doing your best, so you don't need to overdo it.” She had also noticed that she had ignored her own anger to avoid conflicts. The therapist recommended her to try chair work. In the chair work, she could express her anger in an assertive way and she became confident in managing her anger. The therapist also recommended that she write a compassionate letter to be a good friend of her previous self.

### 2.19. In the Tenth Session of CFT

Lisa visited a city centre where she previously worked to obtain a residential certificate. Understandably, she experienced strong anxiety, but her compassionate self and Luffy greatly helped her to gain confidence. She also reported she had soothed her feelings by breathing. She wrote a compassionate letter to say, “I love you so much” to herself.

### 2.20. In the Eleventh Session of CFT

Lisa continued to go to work. She stated that she did not think about her work at all on her days off, even though she had been worried about it before starting CFT. She also reported contacting her old friends, going to the bakery where she worked, and began to reduce the time needed to cope with distress in a compassionate way. The therapist suggested diminishing the frequency of the sessions and discussed this with her. As a result, she hoped to have fortnightly sessions. Even though she sometimes experienced difficulties in relation to colleagues in her present workplace, she stated her confidence in coping with the situation.

### 2.21. In the Twelfth Session of CFT

Lisa had had a quarrel with her grandmother, but she could distance herself from her family at that moment. After that, she could use SRB, and be compassionate for herself and her mother. It was different from her previous experience. She had reported that she could imagine the compassionate self or others immediately and felt less anxious than before.

### 2.22. In the Thirteenth Session of CFT

Lisa stated that she could care for herself no matter what happened. She began not to prolong her distress if she became angry or anxious. About quarrelling with her family (particularly in relation to her grandmother with dementia), she had chosen the right not to be assertive because she thought it was a better and more compassionate way for her. The therapist suggested finishing the therapy and transferring to follow-up sessions and she agreed, although with a degree of loneliness.

### 2.23. Changes in Symptoms

Changes in symptoms are shown in Table 1.

Lisa's depressive symptoms were reduced by the third session and continued to reduce gradually. After the CFT sessions, the scores of SCS-SF had increased greatly and LSAS-J had reduced substantially through the CFT sessions.

## 3. Discussion

We reported a case which was treated by CFT and which was effective for this patient. Her symptoms were reduced by the third session. Even though we did not evaluate symptoms every week, we can infer this reduction relates to psychoeducation of CFT and SRB from session 1 to session 3. The number of studies which have examined the effectiveness of psychoeducation for depression is limited, but it is reported as effective in improving the clinical course, treatment adherence, and psychosocial functioning of depressive patients [26]. Of course, although the psychoeducation of CFT was different from general psychoeducation for major depression, this case can suggest the possible impact of CFT based psychoeducation on depressive symptoms as part of developing a compassionate understanding [25]. From her tears, Lisa seemed to be moved by it and changed her perspective from a self-critical to a compassionate position (session 2). We can also argue for the contribution of SRB in emotion regulation. Lisa had suffered from negative rumination, but she could manage her emotion by using SRB. As a previous report has shown, emotion regulation affects depressive symptoms greatly via more use of rumination and less use of reappraisal [27]. The procedure of CFT which utilizes SRB in the beginning of therapy is a very rational and effective way to regulate emotion. Such emotion regulation and understanding of her status appear to have worked as preparations for conducting compassionate image exercises.

Next, Lisa's compassionate images helped her greatly, particularly after the middle of therapy. Interestingly, she could use different compassionate images depending on the situation. If she needed kindness to help her emotional distress, she used a kind and warm compassionate self. On the other hand, she used compassionate others to ask Luffy to encourage her to overcome her difficulties and this developed her courage. This usage is consistent with the concept of multiple selves that is CFT's core concept of affirming several selves with several emotions or qualities [8]. This concept helps patients to accept and respect the conflicts between different desires and leads them to choose the most adequate way for them. In fact, she could choose a way to inhibit her anger toward her grandmother to avoid unnecessary conflict by considering her desire to get along with her family.

Equally important, she could develop an assertive way because the therapist accepted, understood, and validated her anger. She could not express anger because she could not permit herself to do so. This might be related to her life history and the result of learning; the compassionate attitude of the therapist for her pain encouraged her to express anger toward heartless aggression by a colleague. Such anger is related to shame and social rank which affects various social behaviours and emotions [28]. As she explained that she thought that she was not the same rank as her family because of depression. In the early stage of CFT, social rank is a heavy obstacle to asserting or expressing one's desires, hopes, and emotions. Thus, the therapist's compassionate attitude worked as a safety base to explore ways of emotion expression. Kolts pointed out that the function of the therapist in CFT includes being a safety haven and secure base to explore painful memories or new attempts [25]; thus, we could regard the therapist's compassionate stance and attitudes as a secure base for her, as well as the therapist modelling compassion for her that could then be later internalised. Moreover, her anxiety about social interaction can be considered as a secondary emotion from the perspective of emotion focused therapy [29]. The anxiety was a learned reactive emotion to avoid being considered as defective and which had kept her safe from exposure to social difficulties. To reconstruct such a reactive emotion, shame should be touched within a secure, accepting therapy relationship. The reason the LSAS-J score decreased may be related to such relationships. In other words, we can understand such emotional shift as courage, which is an aspect of compassion. It is inferred that validation and understanding developed her courage that helped her to confront her internal/external difficulties and stop avoidance.

In addition, we can conjecture that the flow of compassion from therapist helped client develop compassion for herself and her mother. The previous report found that compassion from others buffers self-criticism [30] and developing one of three flows (self-to-self, other-to-self, and self-to-others) can activate other two flows of compassion in CFT theory. Lisa had difficulties in experiencing compassion for herself, but she could receive compassion from the therapist. If it seems to be difficult to develop compassion for self, compassion from others via compassionate therapeutic relationships or compassion to others can pave the way to develop compassion for self indirectly. There are useful scales in English to assess the three flows of compassion (Compassion Engagement and Action Scales; CEAS) but the Japanese version has not been developed yet [31]. In clinical work, CEAS would give therapists more meaningful information than SCS-SF.

We can propose the potential effectiveness and feasibility of CFT in Japanese treatment settings, but it is not rooted in particular stand-alone interventions involving compassion. The organization of all CFT components (relationship, psychoeducation, case formulation, and so on) can facilitate the effectiveness of techniques or exercises in developing compassion.

## 4. Conclusion

We introduced a case of CFT of a Japanese patient with repetitive major depression. We illustrated an example which supports the effectiveness of CFT for chronic psychological problems. CFT may be as effective for Japanese people as in the UK, and it could be considered a reliable choice of treatment not only of depressive symptoms but also anxiety.

## Figures and Tables

**Table 1:** Changes in symptoms across treatment.

	Assessment	SS1	SS3	SS5	SS7	SS9	SS11	SS13
BDI-II	32	29	17	7	2	1	0	0
PHQ-9	17	12	5	1	1	1	1	0
SCS-Sf		20						54
Self-compassion		11						25
Self-coldness		27						7
LSAS-J	88							15
